# Methylglyoxal down-regulates the expression of cell cycle associated genes and activates the p53 pathway in human umbilical vein endothelial cells

**DOI:** 10.1038/s41598-018-37937-1

**Published:** 2019-02-04

**Authors:** Jana D. Braun, Diego O. Pastene, Annette Breedijk, Angelica Rodriguez, Björn B. Hofmann, Carsten Sticht, Elke von Ochsenstein, Heike Allgayer, Jacob van den Born, Stephan Bakker, Sibylle J. Hauske, Bernhard K. Krämer, Benito A. Yard, Thomas Albrecht

**Affiliations:** 1 Department of Nephrology, Endocrinology and Rheumatology, Fifth Department of Medicine, Medical Faculty Mannheim, University of Heidelberg, Mannheim, Germany; 2 Center of Medical Research, Medical Faculty Mannheim, University of Heidelberg, Mannheim, Germany; 3 Department of Experimental Surgery - Cancer Metastasis, Medical Faculty Mannheim, University of Heidelberg, Mannheim, Germany; 4 Department of Internal Medicine, University Medical Centre Groningen, Groningen, Netherlands

## Abstract

Although methylglyoxal (MGO) has emerged as key mediator of diabetic microvascular complications, the influence of MGO on the vascular transcriptome has not thoroughly been assessed. Since diabetes is associated with low grade inflammation causing sustained nuclear factor-kappa B (NF-κB) activation, the current study addressed 1) to what extent MGO changes the transcriptome of human umbilical vein endothelial cells (HUVECs) exposed to an inflammatory milieu, 2) what are the dominant pathways by which these changes occur and 3) to what extent is this affected by carnosine, a putative scavenger of MGO. Microarray analysis revealed that exposure of HUVECs to high MGO concentrations significantly changes gene expression, characterized by prominent down-regulation of cell cycle associated genes and up-regulation of heme oxygenase-1 (HO-1). KEGG-based pathway analysis identified six significantly enriched pathways of which the p53 pathway was the most affected. No significant enrichment of inflammatory pathways was found, yet, MGO did inhibit VCAM-1 expression in Western blot analysis. Carnosine significantly counteracted MGO-mediated changes in a subset of differentially expressed genes. Collectively, our results suggest that MGO initiates distinct transcriptional changes in cell cycle/apoptosis genes, which may explain MGO toxicity at high concentrations. MGO did not augment TNF-α induced inflammation.

## Introduction

The incidence of diabetes is rapidly increasing to epidemic proportions, affecting by 2040 1 out of 10 persons globally according to recent estimates^1^. Because diabetes is associated with hyperglycemia-specific micro- and macro-vascular complications, e.g. diabetic nephropathy (DN) and cardiovascular disease, the rapid increase of numbers of people with diabetes will augment the economic costs for morbidity and mortality in coming years thereby absorbing a considerable proportion of the healthcare budget.

For decades, hyperglycemia was considered to be the main driver of late diabetic complications and as such the primary therapeutic target in diabetic patients. Large trials assessing the effect of intensive glycemic control in the general diabetic population^2,3^ have indeed suggested that tighter glycemic control may improve microvascular outcomes in patients with diabetes, yet, the relationship between intensive glycemic control and reduced incidence and/or progression of macro-vascular complications is less clear^4,5^. Even though our understanding of micro- and macro-vascular complications has significantly improved, the therapeutic options for diabetic patients are mostly still limited to blood pressure control, hyperglycemia management, use of a statin and reduction of proteinuria via renin-angiotensin blockade. New therapeutic developments such as SGLT-2 inhibition and GLP-1 agonistic agents, that have recently been shown to improve proteinuria, hold promise to reduce the medical and economic burden associated with DN^6–8^.

The role of oxidative stress as a causal link in the development of hyperglycemia-associated complications has been highlighted in many studies^9,10^. Oxidative stress may cause protein modifications, either directly via reactive oxygen species (ROS), or indirectly by reactive carbonyl products formed by auto-oxidation of carbohydrates, lipids or amino acids. While auto-oxidation of carbohydrates yields precursors of advanced glycation end-products (AGE), e.g. glyoxal, methylglyoxal (MGO) and glycolaldehydes, lipid peroxidation also generates precursors of advanced lipoxidation end-product (ALE), e.g. malondialdehyde (MDA) and 4-hydroxynonenal (4-HNE)^11,12^. AGE and ALE can evoke a variety of biological responses, e.g. stimulation of extracellular matrix production, induction of inflammatory responses and inhibition of proliferation, all of which may perpetuate the progression of diabetic lesions to various degrees^13,14^.

Amongst the precursors of AGE, MGO is a potent glycating agent by far more reactive compared to glucose^15^. It has been suggested that MGO covalently modifies the 20S proteasome^16^ thereby decreasing the ability of diabetic kidneys to eliminate malfunctioning or damaged proteins^17^. Compatible with this suggestion is the finding that knockdown of glyoxalase-1 in non-diabetic mice results in renal lesions indistinguishable from those of diabetic mice, while overexpression of glyoxalase-1 in diabetic mice prevents the development of nephropathy^18^. Other studies have shown that MGO impairs HIF-1α degradation and signaling^19,20^ and activates AMPK mediated autophagic degradation of thioredoxin 1^21^, thus emphasizing its influence on redox homeostasis^22^. Despite the clear association between reactive carbonyl species and diabetic complications, their mode of action on endothelial cells is discussed ambiguously^23–27^. A general finding throughout all studies is however that MGO causes endothelial damage, albeit that different MGO concentrations have been reported at which this occurs^23,28–30^. It is believed that endothelial damage results from apoptosis, yet a comprehensive pathway analysis to our knowledge has not been reported. MGO-mediated apoptosis can be prevented by glycation end-product inhibitors^31,32^, by anti-oxidants^33,34^ and interestingly by cPLA2 inhibition^35^. In the latter study, it also has been suggested that MGO inhibits phosphorylation of nuclear factor-κB (NF-κB) and that pharmacological inhibition of NF-κB further increases MGO-induced apoptosis of human umbilical vein endothelial cells (HUVECs). For a better understanding of MGO-induced cytotoxicity, we assessed to what extent MGO changes the transcriptome of HUVECs exposed to a concurrent inflammatory milieu. In addition, we assessed to what extent this is affected by carnosine (CN), a histidine containing dipeptide with reactive carbonyl scavenging properties.

## Results

### Methylglyoxal significantly alters the gene expression profile

**Figure 1:**
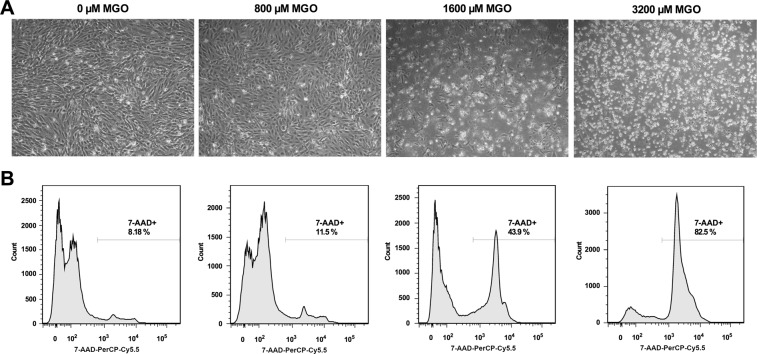
Toxicity of methylglyoxal. HUVECs were exposed to varying concentrations of methylglyoxal (MGO, 0 µM, 800 µM, 1600 µM and 3200 µM) for 24 hours. Changes in cell morphology and toxicity were assessed by phase-contrast microscopy and FACS analysis, respectively. (**A**) Changes in cell morphology (cell detachment, deformation and shrinkage) were apparent at 1600 µM of MGO, while at 800 µM of MGO no changes were observed. (**B**) Dead cells were quantitated by FACS analysis using 7-AAD-PerCP-Cy5.5. While at 0 and 800 µM of MGO the proportion of 7-ADD positive cells was comparable, at 1600 µM of MGO the percentage of 7-AAD positive cells was approximately fourfold higher, i.e. 43.9%. 7-AAD: 7-aminoactinomycin D; MGO: methylglyoxal; PerCP-Cy5.5: peridinin chlorophyll protein cyanine 5.5.

To assess the influence of MGO on gene expression in HUVECs that are exposed to the pro-inflammatory cytokine TNF-α, large-scale gene expression profiling was performed. We first determined susceptibility of HUVECs to MGO by assessing cell viability over a wide range of MGO concentrations (0–3.2 mM). As depicted in Fig. 1, MGO did not change cell morphology at 800 µM while at 1.6 mM cells started to detach accompanied by 7-AAD positive staining in FACS.

**Figure 2:**
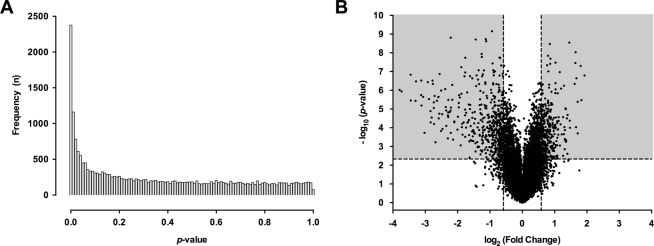
Effect of 800 µM methylglyoxal on gene expression. TNF-α stimulated HUVECS were exposed to 0 µM, 400 µM or 800 µM methylglyoxal (MGO) in the presence or absence of carnosine (CN, 20 mM) for 24 hours. Gene expression profiling was performed by microarray analysis. Displayed are the *p*-value distribution across all examined genes (**A**) and the corresponding volcano plot (**B**) for the comparison 0 µM MGO vs. 800 µM MGO. (**A**) The distribution of *p*-values across all examined genes indicates an excess of *p*-values < 0.05 as suggested by the shape of the histogram. The bars represent a specific number of genes (y-axis) sharing a corresponding *p*-value (x-axis). (**B**) Volcano plot with nominal *p*-values on the y-axis and fold change (FC) expressed as log_2_ on the x-axis. After adjustment for multiple testing (*p*_*adj*_ < 0.05 horizontal line) a total of 2328 genes were found to be significantly affected by 800 µM MGO. After filtering for FC ≥ ±1.5 (Log_2_ (FC) ≤ −0.5 or Log_2_ (FC) ≥ 0.5 vertical lines) 855 transcripts (342 up-regulated and 513 down-regulated) were identified. ANOVA was performed to identify differential expressed genes. A false positive rate of α = 0.05 with false discovery correction was taken as the level of significance.

Since at 800 µM of MGO no obvious toxicity was noted, we used this and a twofold lower MGO concentration for subsequent gene expression profiling. Based on this, in microarray analysis six experimental groups of TNF-α exposed HUVECs were established as follows: no MGO ± CN, 400 µM MGO ± CN and 800 µM MGO ± CN (24 hours incubation). Whereas 400 µM of MGO did not significantly change gene expression compared to no MGO, the transcriptional profile was strongly affected by 800 µM of MGO. The *p*-value distribution on the effect of 800 µM of MGO revealed 4.6 times more genes to be differentially expressed as would be expected if the null hypothesis was true (*p* < 0.0001 by one-tailed binomial test) (Fig. 2A). By applying an adjusted *p*-value < 0.05 (adjusted for multiple testing) and a fold change (FC) threshold of ≥1.5, a total of 855 transcripts (342 up-regulated and 513 down-regulated) were found to be differentially expressed as depicted by the corresponding volcano plot (Fig. 2B).

The ten most down-regulated, respectively up-regulated genes are enlisted in Table 1. As already suggested by the skewing of the volcano plot to the left (more genes, higher significance and larger FC), the MGO mediated change in gene expression profile was dominated more by down-regulation than by up-regulation of gene expression. Amongst the up-regulated genes heme oxygenase-1 (*HO-1*) was ranked the highest with an approximately 3.8 fold increased expression (*p* = 9.63E-5). Down-regulated genes mostly involved cell cycle dependent genes, e.g. topoisomerase (DNA) II alpha (*TOP2A*), abnormal spindle microtubule assembly (*ASPM*), kinesin family member 20 A (*KIF20A*), marker of proliferation Ki-67 (*MKI67*) and cyclin A2 (*CCNA2*) (all *p* < 0.001) (Table 1).

**Table 1:** List of top twenty most differentially expressed genes between 800 µM MGO and Control (*p* < 0.05, fold change ≥ ±1.5).

Gene symbol	Gene name	Fold change	*p*-value
800 µM MGO × Control: Up-regulated			
HO-1	heme oxygenase 1	3.79	9.63E-5
PSAT1	phosphoserine aminotransferase 1	3.55	5.93E-4
DKK1	dickkopf WNT signaling pathway inhibitor	3.49	5.40E-5
VTRNA1-3	vault RNA 1-3	3.28	0.00620
PTGS2	prostaglandin-endoperoxide synthase 2	3.27	0.00068
LPXN	leupaxin	3.19	0.00012
SERPINB2	plasminogen activator inhibitor 2	3.17	0.00420
ATP1B1	ATPase Na+/K+ transporting subunit beta 1	3.14	2.90E-5
MARCH4	membrane associated ring-CH-type finger 4	3.12	5.04E-5
IGFBP3	insulin like growth factor binding protein 3	3.07	0.00018
**800** **µM MGO × Control: Down-regulated**			
TOP2A	topoisomerase (DNA) II alpha	−13.88	0.00045
ASPM	abnormal spindle microtubule assembly	−13.30	0.00030
KIF20A	kinesin family member 20a	−10.96	9.58E-5
MKI67	marker of proliferation Ki-67	−10.92	0.00061
FABP4	fatty acid binding protein 4, adipocyte	−9.85	0.00062
BUB1	BUB1 mitotic checkpoint serine/threonine	−9.76	0.00016
TTK	TTK protein kinase	−8.74	0.00039
CASC 5	cancer susceptibility candidate 5	−8.72	0.00014
CCNA2	cyclin A2	−8.67	0.00085
DLGAP5	DLG Associated Protein 5	−8.38	0.00045

### Enriched genes cluster in the categories cell cycle, mitosis and apoptosis

**Figure 3:**
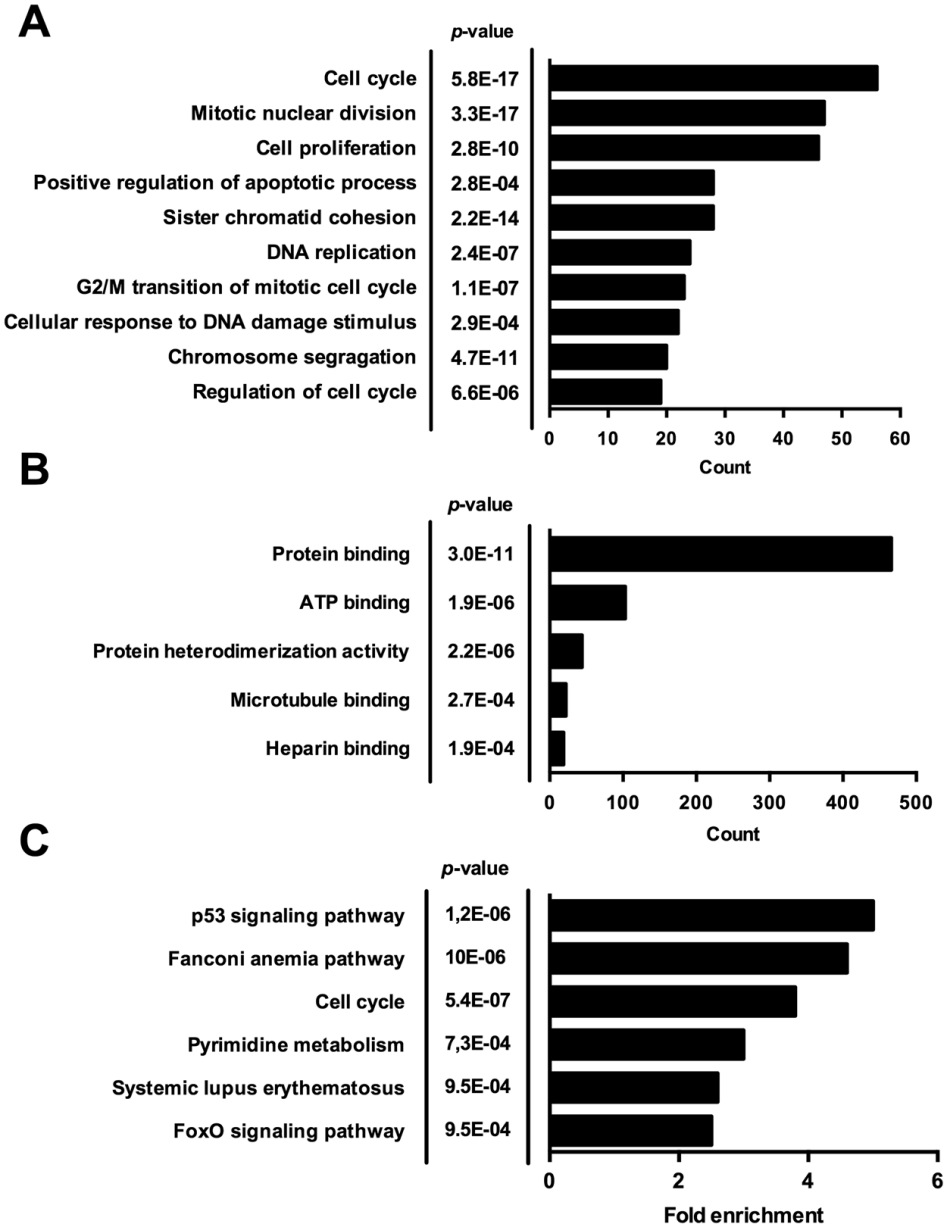
Differential gene expression by 800 µM methylglyoxal is compatible with cell cycle disruption and apoptosis. TNF-α stimulated HUVECS were exposed to 0 µM, 400 µM or 800 µM methylglyoxal (MGO) in the presence or absence of carnosine (CN, 20 mM) for 24 hours. Gene expression profiling was performed by microarray analysis. All differentially expressed genes (DEG, *p*_adj_ < 0.05, FC ≥ ±1.5 (up or down)) in the comparison 0 µM vs. 800 µM MGO were subjected to Gene Ontology (A and B) and KEGG pathway analysis (**C**) using DAVID (version 6.8, https://david.ncifcrf.gov/). (**A**) GO biological process analysis of all differentially expressed genes revealed ten biological process categories to be significantly enriched, most of which related to cell cycle. (**B**) GO molecular function analysis of all DEGs showed that the majority of the corresponding proteins were annotated a protein binding function. (**C**) KEGG-based pathway analysis of all DEGs identified six pathways to be significantly enriched. The *Benjamini-Hochberg* procedure was applied in functional annotation and pathway analysis to account for multiple hypothesis testing as provided by DAVID. Reported are the adjusted *p*-values.

All 855 differentially expressed genes (DEGs) were subjected to Gene Ontology (GO) analysis. In the GO Biological Process category, the top five overrepresented annotations were cell cycle (56 genes, *p* = 5.8E-17), mitotic nuclear division (47 genes, *p* = 3.3E-17), cell proliferation (46 genes, *p* = 2.8E-10), positive regulation of apoptotic process (28 genes, *p* = 2.8E-4) and sister chromatid cohesion (28 genes, *p* = 2.2E-14) (Fig. 3A). In the GO Molecular Function category (Fig. 3B), most of the DEGs were annotated a protein binding function (466 genes, *p* = 3.0E-11), by far outweighing other functions such as ATP (103 genes, *p* = 1.9E-6) or microtubule binding (22 genes, *p* = 2.7E-4). Functional clustering using the functional annotation clustering tool provided by DAVID (set on high classification stringency) identified one strongly overrepresented annotation group defined by the terms “mitosis” and “cell division” with an enrichment score of 22.08.

In addition, we performed pathway analysis based on the KEGG database. Using a Benjamini-corrected *p*-value threshold of 0.05, six significantly enriched pathways were identified with the p53 signaling pathway being the most affected (fold enrichment 5.0, *p* = 1.2E-6; Fig. 3C), followed by the Fanconi anemia pathway (*p* = 10E-6), Cell cycle (*p* = 5.4E-7), Pyrimidine metabolism (*p* = 7.3E-4), Systemic lupus erythematosus (*p* = 9.5E-4) and the Forkhead box O (*FoxO*) signaling pathway (*p* = 9.5E-4).

Using *p*_*adj*_ < 0.05 and FC ≥ ±1.5 as selection criteria, 15 genes in the p53 signaling pathway were significantly affected by MGO (Table 2). Seven out of these are generally believed to be inversely associated with p53 activation: cyclin-dependent kinase 1 (*CDK1*), cyclin B2 (*CCNB2*), cyclin B1 (*CCNB1*), ribonucleotide reductase regulatory subunit M2 (*RRM2*), G-2 and S-phase expressed 1 (*GTSE1*), cyclin G2 (*CCNG2*) and checkpoint kinase 1 (*CHEK1*). Consistent with activation of the p53 pathway, all seven genes were strongly down-regulated by MGO with fold changes as high as −7.40 for *CDK1* or −6.02 for *CCNB2*. The remaining eight selected genes either directly act as mediators of apoptosis, growth arrest or as upstream p53 activators: insulin-like growth factor binding protein 3 (*IGFBP3*), sestrin 2 (*SESN2*), growth arrest and DNA damage inducible beta (*GADD45B*), Fas cell surface death receptor (FAS), phorbol-12-myristate-13-acetate-induced protein (*PMAIP1*), p21 (*CDKN1A*), ATM serine/threonine kinase (*ATM*) and sestrin 3 (*SESN3*). With the exception of *SESN3* and *ATM*, these genes were up-regulated by MGO. Hence, our data demonstrate that out of 15 genes affected by MGO in the p53 pathway the expression of 13 changed in a direction that would be expected upon p53 activation.

**Table 2:** Significantly enriched genes of the p53 signaling pathway (*p* < 0.05, fold change ≥ ±1.5).

Gene symbol	Gene name	Function^a^	Fold change
800 µM MGO × Control: p53 pathway			
CDK1	cyclin dependent kinase 1	repressed upon p53 activation	−7.40
CCNB2	cyclin B2	repressed upon p53 activation	−6.02
CCNB1	cyclin B1	repressed upon p53 activation	−5.22
RRM2	ribonucleotide reductase regulatory subunit M2	repressed upon p53 activation	−4.41
GTSE1	G-2 and S-phase expressed 1	negative regulator of p53	−3.20
SESN3	sestrin 3	p53 mediator^b^	−2.34
CCNG2	cyclin G2	negative regulator of p53	−1.71
ATM	ATM serine/threonine kinase	activator of p53	−1.64
CHEK1	checkpoint kinase 1	repressed upon p53 activation	−1.51
CDKN1A	cyclin dependent kinase inhibitor 1A (p21)	p53 mediator of growth arrest	+1.50
PMAIP1	phorbol-12-myristate-13-acetate-induced protein (noxa)	p53 mediator of apoptosis	+1.54
FAS	Fas cell surface death receptor	p53 mediator of apoptosis	+1.55
GADD45B	growth arrest and DNA damage inducible beta	p53 mediator of apoptosis	+1.96
SESN2	sestrin 2	p53 mediator of growth arrest	+1.97
IGFBP3	insulin like growth factor binding protein 3	p53 mediator of apoptosis	+3.07

^a^Function derived from current literature.

^b^Only sestrin 1 and 2 are direct transcriptional targets of p53. Sestrin 3 is primarily activated by the FoxO family.

### Influence of carnosine on MGO-induced differential gene expression

**Figure 4:**
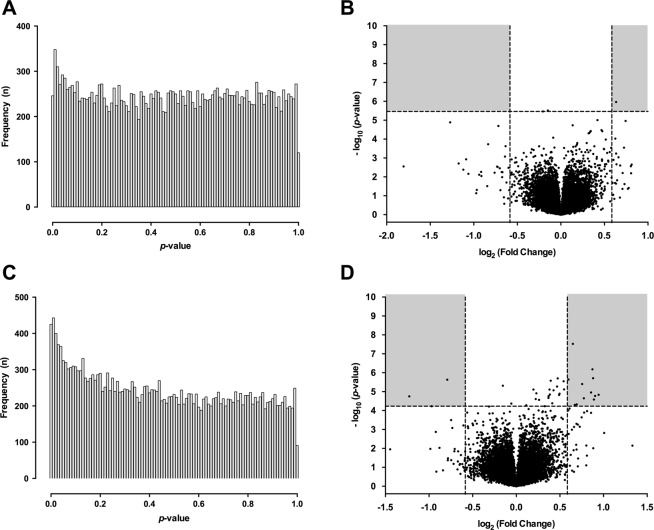
Effect of carnosine on gene expression. TNF-α stimulated HUVECS were exposed to 0 µM, 400 µM or 800 µM methylglyoxal (MGO) in the presence or absence of carnosine (CN, 20 mM) for 24 hours. Gene expression profiling was performed by microarray analysis. Displayed are the *p*-value distribution across all examined genes (**A**,**C**) and the corresponding volcano plot (**B** and **D**) for the comparisons 0 µM MGO no CN vs. 0 µM MGO + 20 mM CN (upper panel) and 800 µM MGO no CN vs. 800 µM MGO + 20 mM CN (lower panel). (**A**) The *p*-value distribution across all genes shows a homogenous pattern indicating that CN did not significantly affect the gene expression profile of TNF-α stimulated HUVECs. (**B**) The corresponding volcano plot analysis revealed only three genes to be significantly affected by CN as compared to control. (**C**) The *p*-value distribution shows a relative excess of *p*-values < 0.05, though this effect was less pronounced as compared to the *p*-value distribution in Fig. 3A. (**D**) The corresponding volcano plot analysis revealed 37 genes to be significantly affected by CN. Multiple hypothesis testing was accounted for by application of false discovery correction. The significance threshold for adjusted *p*-values in all analyses was defined as 0.05, which corresponds to the nominal significance thresholds represented by the horizontal lines in B and D. The different nominal threshold values result from the experiment-dependent multiplicity adjustment. Vertical lines represent thresholds for FC ≥ ±1.5 (Log_2_ (FC) ≤ −0.5 or Log_2_ (FC) ≥ 0.5).

We next assessed if CN, a scavenger of reactive carbonyl species, is able to counteract MGO-induced gene expression. In the absence of MGO, CN did not significantly affect gene expression (no CN vs. 20 mM CN) (Fig. 4A,B). Although addition of CN to MGO-stimulated HUVECs had a significant effect on the transcriptome with approximately 1.8 times more genes affected than what would be expected if the null hypothesis were true (*p* < 0.0001 by one-tailed binomial test, CN + 800 µM MGO vs. 800 µM MGO) (Fig. 4C,D), this was clearly less strong as compared to the effect of MGO *per se*, i.e. MGO vs. no MGO (Fig. 2).

**Figure 5:**
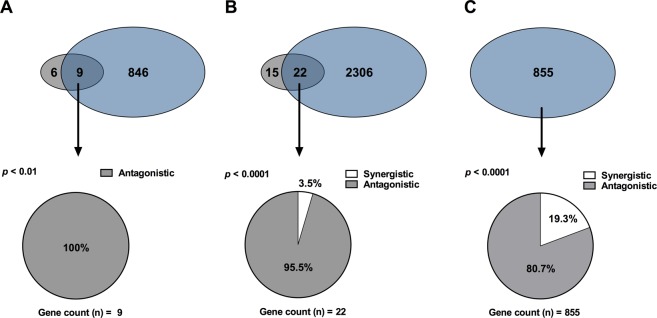
Influence of carnosine on MGO-induced differential gene expression. TNF-α stimulated HUVECS were exposed to 0 µM, 400 µM or 800 µM methylglyoxal (MGO) in the presence or absence of carnosine (CN, 20 mM) for 24 hours. Gene expression profiling was performed by microarray analysis. All pie charts depict, how many of a selected number of genes are synergistically/antagonistically regulated genes by CN + 800 µM MGO vs. only 800 µM MGO (both in presence of TNF-α). (**A**) The upper Venn-diagram displays differentially expressed genes (DEG, *p*_adj_ < 0.05, FC ≥ ±1.5 (up or down)) for the comparisons 800 µM MGO vs. 800 µM MGO + 20 mM CN and 0 µM MGO vs. 800 µM of MGO. The commonly shared differences (overlap) were further analyzed (arrow) and depicted as pie charts for CN counteracting the effect of MGO (antagonistic) or displaying a similar effect as MGO (synergistic). Note that all commonly shared differences found in the upper Venn-diagram were counteracted by CN (*p* < 0.01). (**B**) The upper Venn-diagram displays differentially expressed genes (DEG, *p*_adj_ < 0.05 irrespective of the fold change) for the comparisons 800 µM MGO vs. 800 µM MGO + 20 mM CN and 0 µM MGO vs. 800 µM of MGO. The commonly shared differences (overlap) were further analyzed (arrow) and depicted as pie charts for CN counteracting the effect of MGO (antagonistic) or displaying a similar effect as MGO (synergistic). Note that 95.5% of the commonly shared differences were counteracted by CN (*p* < 0.001). (**C**) The upper Venn-diagram displays differentially expressed genes (DEG, *p*_adj_ < 0.05, FC ≥ ±1.5 (up or down)) for the comparison 0 µM MGO vs. 800 µM of MGO only. The proportion of DEGs that was counteracted by CN in quantitative terms is displayed as pie chart. Note that 80.7% of the genes were regulated antagonistically (*p* < 0.0001). One-tailed binomial test was calculated to compare observed and expected frequencies.

After applying the *p*_*adj*_ < 0.05 and FC ≥ ±1.5 selection criteria, 15 genes remained significant (12 were up-regulated, 3 were down-regulated, Table 3). As depicted in Fig. 5A, 9 of these 15 genes were also differentially expressed by MGO: aldehyde dehydrogenase 1 family member A1 (*ALDH1A1)*, collagen type XII alpha 1 (*COL12A1*), diaphanous related formin 2 (*DIAPH2*), ATPase class I type 8B member 1 (*ATP8B1*), nidogen 2 (*NID2*), endomucin (*EMCN*), arylacetamide deacetylase pseudogene 1 (*AADACP1*), leupaxin (*LPXN*) and heme oxygenase-1 (*HO-1*). Importantly, the expression levels of all 9 genes were inversely regulated by CN relative to the effect of MGO (*p* < 0.01 by one-tailed binomial test, Fig. 5A). This observation remained if a less stringent DEG definition was applied, including those genes with a *p*_*adj*_ < 0.05 irrespective of the fold change. Using these criteria, out of the 37 genes changed by carnosine, 22 were also differentially expressed by MGO. Importantly, 21 of these 22 genes were inversely regulated by CN (95.5%, *p* < 0.0001 by one-tailed binomial test, Fig. 5B). Even if only MGO-mediated DEGs were considered (whether or whether not the effect of carnosine was significant), the number of inversely regulated genes (690 genes, 80.3%) significantly outbalanced those that were concordantly regulated (165 genes, 19.3%, *p* < 0.0001 by one-tailed binomial test, Fig. 5C). Hence, even though the number of MGO affected genes that were counteracted by CN was relatively small when stringent selection criteria were applied (*p*_*adj*_ < 0.05 and FC ≥ ±1.5), most of MGO-mediated changes were inversely regulated by carnosine in quantitative terms. This also held true, when the comparison was restricted to the MGO-mediated DEGs involved in the p53 pathway and cell cycle (80% and 73% reciprocal expression by carnosine, respectively).

**Table 3:** List of most differentially expressed genes between 800 µM MGO + Carnosine and 800 µM MGO (*p* < 0.05, fold change ≥ ±1.5).

Gene symbol	Gene name	Fold change	*p*-value
800 µM MGO + CN × 800 µM MGO: Up-regulated			
FBXO32	F-box protein 32	1.92	0.01772
ALDH1A1	aldehyde dehydrogenase 1 family member A1	1.87	0.01819
PLCB1	phospholipase C, beta 1	1.85	0.02289
GPX8	glutathione peroxidase 8	1.84	0.00876
COL12A1	collagen, type XII, alpha 1	1.83	0.00814
DIAPH2	diaphanous related formin 2	1.81	0.01711
PDE7B	phosphodiesterase 7B	1.71	0.02137
ATP8B1	ATPase, class I, type 8B, member 1	1.69	0.01092
UACA	uveal autoantigen with coiled-coil domains and ankyrin repeats	1.62	0.03662
NID2	nidogen 2 (osteonidogen)	1.59	0.03754
ADGRF5	adhesion G protein-coupled receptor F5	1.57	0.00074
EMCN	endomucin	1.51	0.00876
**800** **µM MGO** + **CN × 800** **µM MGO: Down-regulated**			
HO-1	heme oxygenase 1	−2.34	0.01819
LPXN	leupaxin	−1.96	0.04058
AADACP1	arylacetamide deacetylase pseudogene 1	−1.73	0.00876

### Methylglyoxal suppresses TNF-α driven VCAM-1 expression

**Figure 6:**
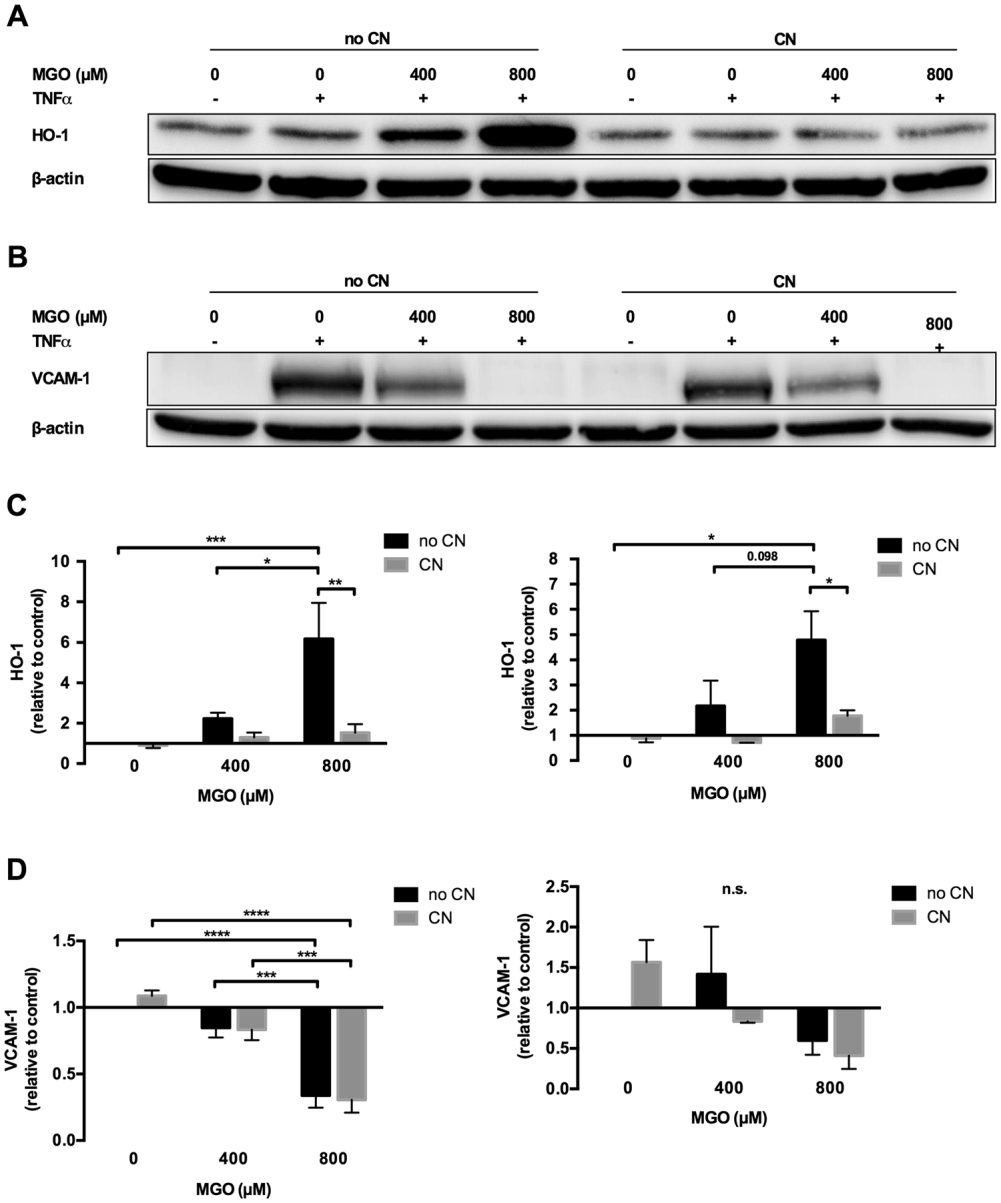
Methylglyoxal increased HO-1 expression, but diminished TNF-α induced VCAM-1 expression. HUVECs were stimulated with different combinations of methylglyoxal (MGO, 0 µM, 400 µM and 800 µM), TNF-α (12.5 ng/ml) and carnosine (CN, 20 mM). Western blotting and qPCR regarding HO-1 and VCAM-1 were performed after protein and RNA isolation. (**A**) HO-1 Western blotting demonstrates dose-dependent increase of HO-1 expression by MGO, which was abrogated by CN. (**B**) VCAM-1 Western blotting shows a clear TNF-α induced increase of the negligible baseline VCAM-1 signal, which was dose-dependently reduced by MGO. Equal protein loading was demonstrated by staining for β-actin. Displayed are the cropped blots. (**A**,**B**) show two independent experiments. The black surrounding lines demarcate individual blots. The scanned full-length blots are provided in Supplementary Figs 1 and 2. (**C**) HO-1 densitometry (figure to the left) and gene expression (figure to the right) show a significant and dose dependent increase of HO-1 quantity, which was significantly reversed by CN. Protein and mRNA expression were normalized to β*-*actin. (**D**) VCAM-1 densitometry (figure to the left) and gene expression (figure to the right) demonstrate a significant, dose-dependent reduction of VCAM-1 by MGO at the protein, but not at the mRNA level. Carnosine did not significantly affect VCAM-1 quantity. Protein and mRNA expression were normalized to β*-*actin. For C and D data were analyzed using two-way ANOVA followed by Turkey’s multiple correction test. A *p*-value < 0.05 was considered to be significant. *p < 0.05; **p < 0.01; ***p < 0.001; ****p < 0.0001.

Western blotting of cells stimulated with 12.5 ng/ml TNF-α in the absence of MGO showed, that TNF-α exposure alone did not affect HO-1 or VCAM-1 protein levels. In concordance with the gene expression profiling data, addition of MGO, however, dose-dependently up-regulated HO-1 protein and mRNA expression (Fig. 6A,C). Interestingly, VCAM-1 protein but not VCAM-1 mRNA was significantly and reproducibly (n = 3) down-regulated upon addition of MGO (Fig. 6B,D). While addition of CN clearly diminished MGO-mediated HO-1 up-regulation, it did not affect VCAM-1 expression (Fig. 6A–D). In line with these findings, also Affymetrix analysis revealed no significant change in VCAM-1 mRNA upon MGO exposure.

## Discussion

Current data on the mechanisms by which MGO instigates vascular injury are inconsistent and it is questionable to what extent inflammation contributes to this. The present study was designed to assess the influence of MGO on the vascular transcriptome of HUVECs exposed to a concurrent inflammatory milieu. Although MGO has been subject of many *in vitro* and *in vivo* studies, to our knowledge this is the first study that in a detailed and comprehensive manner assessed the effects of MGO on gene expression in a type of human endothelial cells. For this purpose, microarray analysis was performed on six different groups of TNF-α stimulated HUVECs, that were exposed to a concentration of either 0 µM, 400 µM or 800 µM MGO in the presence or absence of carnosine for a period of 24 hours. Our findings indicate that MGO severely impairs the expression of cell cycle associated genes and leads to a gene expression signature that is compatible with activation of the p53 pathway. CN inversely regulated most of the transcriptional changes induced by MGO in quantitative terms, albeit that this was only found for a small subset of genes when stringent selection criteria were applied (*p*_*adj*_ < 0.05 and FC ≥ ±1.5).

High MGO concentrations inflict a strong biological effect on transcriptional activity in HUVECs as can be appreciated from the shape of the *p*-value distribution histogram (Fig. 2A). Amongst the genes that were up-regulated by MGO, HO-1 was ranked the highest on the basis of FC and *p*-value. How MGO regulates HO-1 expression mechanistically was not addressed, yet this likely involves the transcription factor nuclear factor erythroid-2-related factor 2 (*Nrf-2*) system as suggested by a concordant up-regulation of other Nrf-2 target genes, i.e. phosphoserine aminotransferase 1 (*PSAT1*) (FC: 3.55, *p*_*adj*_ = 5.93E-4, Table 1)^36^ and NAD(P)H dehydrogenase, quinone 1 (*NQO-1*, FC: 2.44, *p*_*adj*_ = 0.0001, data not shown). In keeping with the many studies that have elaborated on the tissue protective properties of the Nrf-2 system, our finding of MGO-induced HO-1 expression in HUVECs is unexpected and unprecedented. Nonetheless as an opposing view to the majority of studies, it should also be mentioned that a recent study suggested HO-1 to be a driver of meta-inflammation and insulin resistance in mouse and man^37^.

Our data set of DEGs also includes genes, which previously have been reported to be relevant players in T2D patients and in *in vivo* diabetic models. DKK1 seems to promote the development of DN in diabetic rats^38^ and in humans circulating DKK1 levels are increased in type 2 diabetic patients^39^. Circulating concentrations of IGFI and IGFBP3 have been associated with risk of type 2 diabetes in epidemiological studies^40,41^ and IGFBP3 has been shown to cause insulin resistance independently of IGF binding in adipocytes^42^.

The most pronounced effect of MGO related to down-regulation of cell cycle associated genes. GO analysis of the DEGs showed significant accumulation in biological processes related to cell cycle, mitosis and apoptosis. By making use of the KEGG database, the p53 pathway was found to be the most enriched. Analysis of the function of the corresponding enriched genes and their direction of change revealed that 13 out of 15 genes changed in a direction that would be expected upon p53 activation.

Previously, *Hsieh et al*. showed that serpinB2 is a direct downstream target of p53. They also showed that serpinB2 binds and stabilizes p21 to mediate senescence independent of its extracellular function to inhibit urokinase-type and tissue-type plasminogen activator (uPA and tPA), respectively^43^. In our study, serpinB2 was amongst the top 10 genes that were upregulated by MGO (Table 1, FC: 3.17; *p*_*adj*_ = 0.0042). Since p21 (*CDKN1A*), also a downstream target of p53, is a cell cycle inhibitor, the increased expression of serpinB2 by MGO may likely sustain cell cycle arrest upon MGO treatment.

Functional clustering and pathway analysis also revealed significant enrichment of the FoxO pathway. Activation of the FoxO pathway promotes cell cycle arrest at the G1/S boundary by up-regulation of cell cycle suppressor genes such as p27 and mediates apoptosis both through the intrinsic and extrinsic apoptotic pathway^44^. The p53 and FoxO pathways may orchestrate apoptosis and growth arrest in a concerted action through sharing of common targets^45^. Collectively, gene set enrichment analysis was compatible with a dysregulation of the regular cell cycle progression and activation of apoptosis pathways.

The mechanism underlying MGO-induced effects on gene expression most likely includes carbonylation of transcription factors or signaling molecules such as ERK, JNK and p38 MAPK since addition of aminoguanidine prevents MGO mediated MAPK activation^29^. The latter observation is in line with our findings that the MGO scavenger CN also mitigates the effect of MGO on gene expression.

While MGO mediated cell death has been reported to be caspase-independent and may have features of necrosis as reported previously^28,46^, our findings are in line with most studies identifying apoptosis as the predominant mode by which MGO causes cell death^47–50^. MGO-induced cell death is widely considered to be associated with inflammation as evidenced by increased levels of inflammatory mediators^23,51,52^, which is strongly reduced in diabetic mice overexpressing glyoxalase-1^52^. Yet, MGO may also inhibit NF-kappa B p65 DNA-binding in a site-specific manner favoring cell death upon TNF-α stimulation^53^. With exception of cyclooxygenase-2 (*PTGS2*) up-regulation (Table 1, FC: 3.27, *p*_*adj*_ = 0.00068), we did not observe an inflammation augmenting effect of MGO when assessing gene enrichment in pathways that are primarily implicated in inflammation or immune responses. On contrary, MGO reduced the expression of TNF-α induced VCAM-1, which was hardly detectable at baseline (i.e. no TNF-α) as has been shown in previous *in vitro* studies^54–56^. This was an unexpected observation as neither gene expression profiling, nor qPCR revealed a significant change in VCAM-1 mRNA expression. These findings therefore suggest that down-regulation of VCAM-1 protein most likely occurs post-transcriptionally. Regulation of VCAM-1 expression may occur by inhibition of its translation through the interaction of miR-126 with the three prime untranslated region (3′-UTR) of the VCAM-1 mRNA^57^ or by controlling its mRNA stability^58,59^. Alternatively, MGO may increase proteasomal degradation by glycation-induced protein ubiquitination in a similar manner as has been demonstrated for other proteins^60,61^. Our findings question the pro-inflammatory effect of MGO, however, they are not sufficient to claim an anti-inflammatory property of MGO, in particular since inflammatory pathways were not significantly altered in the microarray analysis.

This is the first study to show that the dipeptide CN is capable to reverse MGO-induced effects on gene expression to a large extent. This was not only observed when stringent selection criteria were applied (*p*_*adj*_ < 0.05 and FC ≥ ±1.5). Also, when allowing a smaller FC the majority of genes that were differentially expressed in the comparison TNF-α vs. TNF-α + MGO were inversely regulated in the comparison TNF-α + MGO vs. TNF-α + MGO + CN. We therefore postulate, that the mechanisms by which CN mediates these effects are either through inactivation of the reactive nature of MGO or alternatively by preventing cellular uptake of MGO.

This study has a few limitations to be considered. First of all, the results obtained are solely based on *in vitro* experiments. Yet, designed as a mechanistic study this work intended to dissect the toxic effects of reactive carbonyl species like MGO on the vasculature. For such purposes, a cell culture based approach with the use of one specific cell type (HUVECs), which also allows optimal controllability of experimental conditions seems appropriate. We believe that the obtained results provide a robust fundament to both further evaluate the systemic effects of MGO on diabetes-rendered organisms, e.g. by administration of MGO in rodents, and to assess the therapeutic potential of CN in this context. As elucidated by the affected pathways, this study also provides evidence to further evaluate new treatments that may confer anti-apoptotic effects as it has been shown for GLP-1^62,63^.

Secondly, though HUVECs by far represent the most widely used cell type to study endothelial dysfunction in diabetes, the results obtained herein cannot be easily transferred to other types of endothelial cells (e.g. microvascular) that are implicated in diabetic complications. Therefore, it should be emphasized that our finding should not be generalized as a number of studies have demonstrated significant heterogeneity in endothelial structure and function^64–67^.

Finally, the MGO concentrations used in this study are relatively high and by far exceed MGO concentrations reported in plasma of healthy individuals or patients with diabetes. Although this makes translation of our findings to *in vivo* difficult, it should be underscored that *in vivo* other factors might work in synergy with MGO to cause vascular damage. The applied MGO concentrations (400 and 800 µM) were chosen on the basis of dose-response analyses with cell toxicity as read-out parameter. As such, this study is in line with previous *in vitro* studies that used similar MGO concentrations to inflict injury to endothelial cells^23,24,31,68–71^. Since the intention of the current study was to determine to what extent MGO changes the transcriptome at a near toxic concentration and whether CN was able to overcome these changes, the aforementioned MGO concentrations were essential.

In conclusion, we have shown that MGO significantly alters the gene expression profile of TNF-α stimulated HUVECs, most strongly affecting cell cycle progression and activation of apoptotic pathways. We also demonstrated, that CN counteracts MGO-induced differential gene expression to a considerable extent underscoring its potential use in the treatment or prevention of diabetic complications. This study questions the pro-inflammatory properties of MGO and warrants further *in vitro* and *in vivo* studies to re-evaluate its impact on inflammation in diabetic conditions.

## Methods

### Cell Culture

HUVECs were isolated from fresh umbilical cords. The cells were cultured on 1% gelatin coated flasks (Fluka, Neu-Ulm, Germany) in endothelial cell growth medium (Provitro, Berlin, Germany) supplemented with essential growth factors and 5% fetal bovine serum (Gibco, Carlsbad, USA). Cultures were maintained at 37 °C, 95% relative humidity and 5% CO_2_. Confluent monolayers were passaged by trypsin 0.025%/EDTA 0.01% and experiments were conducted on cells in passage 2–6 at approximately 90–100% confluence. Um WT Plus Reagent bilical cords were obtained from the Department of Obstetrics, University Medical Center Mannheim after written informed consent. The study was approved by the local ethics committee (Medizinische Ethikkommission II der Medizinischen Fakultät Mannheim (No. 2015–518N-MA)) and carried out in accordance with the relevant guidelines and regulations.

HUVECs were characterized by means of indirect immune fluorescence and FACS analysis. To this end, cells were grown on gelatin coated coverslips until confluence. The cells were fixed by ice cold methanol, thoroughly washed with PBS and subsequently incubated with the primary antibodies von Willebrand Factor (Dako, Wiesentheid, Germany) and ZO-1 (Santa Cruz, Heidelberg, Germany) and the appropriate secondary antibodies Texas Red-X (Goat anti-Mouse IgG, Invitrogen, Karlsruhe, Germany) and IgG Alexa Fluor 488 (Donkey anti-Rabbit, Invitrogen). Cells were visualized by fluorescence microscopy (Supplementary Fig. 3). FACS analysis was performed according to standard procedures using directly conjugated anti-CD31-APC antibodies as marker. The cells were analyzed on a FACS lyrics platform (BD, Heidelberg, Germany). Based on CD31 expression an endothelial cell purity of >98% was generally achieved (Supplementary Fig. 3).

For the main experiments, HUVECs were treated with different substances as indicated: 12.5 ng/ml TNF-α (PeproTech, Hamburg, Germany), 50–800 µM MGO (Sigma-Aldrich, St. Louis, USA) and 20 mM L-carnosine (Sigma-Aldrich).

### RNA Isolation and microarray analysis

Total RNA was prepared using TRIzol reagent (Ambion, Carlsbad, USA). RNA quality was confirmed by capillary electrophoresis on an Agilent 2100 bioanalyzer. For the microarray experiments six T25 flasks of HUVEC were stimulated with TNF-α (12.5 ng/ml), to which different concentrations of MGO were added in the presence or absence of 20 mM of L-carnosine (CN) for 24 hours as described in the following:

Flask 1: TNF-α, no MGO, no CN

Flask 2: TNF-α, no MGO, 20 mM CN

Flask 3: TNF-α, 400 µM MGO, no CN

Flask 4: TNF-α, 400 µM MGO, 20 mM CN

Flask 5: TNF-α, 800 µM MGO, no CN

Flask 6: TNF-α, 800 µM MGO, 20 mM CN

Gene expression profiling was performed using arrays of human HuGene-2_0-st-type from Affymetrix (Santa Clara, USA). Biotinylated antisense cRNA was then prepared according to the Affymetrix standard labelling protocol with the GeneChip® WT Plus Reagent Kit and the GeneChip® Hybridization, Wash and Stain Kit (both from Affymetrix). Afterwards, chip hybridization was performed on a GeneChip Hybridization Oven 640, then dyed in the GeneChip Fluidics Station 450 and thereafter scanned with a GeneChip Scanner 3000. All of the equipment used was purchased from Affymetrix.

### Quantitative PCR

For qPCR, the groups were employed as described above. To obtain total RNA, the cells were homogenized in TRIzol reagent (Ambion). Before reverse transcription, isolated RNA was treated with RNase free DNase I (Ambion). 1 µg of total RNA was reversed-transcribed into cDNA using the High-Capacity cDNA Reverse Transcription Kit (Applied Biosystems, Foster City, USA) according to the manufacturer’s instructions and diluted in 20 µl nuclease-free water (Ambion). Quantitative PCR was performed on a 7900HT Real-Time PCR System (Applied Biosystems) using TaqMan Fast Advanced Master Mix (Applied Biosystems) and the following TaqMan probes (Applied Biosystems): HMOX1 (HO-1, ID: Hs 01110250_m1), VCAM1 (ID: Hs 01003372_m1) and ACTB (β-actin, ID: Hs 01060665_g1). The following thermal cycling profile was used for all samples: 2 min at 50 °C, 20 sec at 95 °C followed by 40 cycles of 1 sec at 95 °C and 20 sec at 60 °C. All samples were normalized for an equal expression of β-actin and the depicted results are relative to the control group.

### Protein isolation and Western blotting

For Western blotting, in addition to the groups described above a control group with only medium (no TNF-α) was employed. Hereafter, proteins were isolated as follows: After one wash step with PBS (Gibco), HUVECS were lysed on ice using lysis buffer supplemented with dithiothreitol (Invitrogen, Carlsbad, USA), protease inhibitor (Roche, Indianapolis, USA), and phosphatase inhibitor (Sigma-Aldrich). In order to remove cell debris, cell lysates were centrifuged for 10 minutes at 12000 G and 4 °C. Protein concentrations were measured using Bio-Rad Protein Assay Kit. All samples (20 µg of total protein) were 3:1 diluted in 4x Laemmli sample buffer (Bio-Rad, Hercules, USA) and heated up to 100 °C for 5 minutes to denature proteins. The samples were then loaded on a 10% SDS-PAGE and transferred to a PVDF membrane by semi-dry blotting. Membranes were blocked in TBS (Bio-Rad) containing 0.1% Tween (Sigma-Aldrich) and 5% milk powder for 1 hour at room temperature. Anti-VCAM-1 (R&D Systems, Wiesbaden, Germany) and anti-HO-1 (Enzo, Farmingdale, USA) were used as the first antibody. After incubation with the appropriate horseradish-peroxidase-conjugated secondary antibodies, proteins were visualized by chemiluminescence according to the manufacturer’s instructions. Equal protein loading was confirmed by β-actin intensity using specific antibodies (Santa Cruz Biotechnology, Dallas, USA). Protein expression was quantitated by densitometry and normalized by β*-*actin levels.

### FACS

HUVECs were resuspended from T25 flasks at confluence, centrifuged (300 G, 9 minutes, room temperature), and washed two times with 2 mL cold PBS (1500 rpm, 5 minutes, room temperature). Cell pellets were then resuspended in 100 µl PBS and stained with 5 µl 7-Amino-Actomicyin D (7-AAD) for dead cell exclusion analysis (BD-Biosciences, incubation in dark environment for 20 minutes). Finally, cells were washed three times with 1 ml cold PBS (1500 rpm, 5 minutes, room temperature), resuspended in 200 µl PBS, and measured in a FACS-Canto II flow cytometer (Diva Software) within 30 minutes. Results were post-processed with FlowJo software version 5.2 (FlowJo LLC, Ashland, USA).

### Statistical analysis

A Custom CDF Version 20 with ENTREZ based gene definitions was used to annotate the arrays. The raw fluorescence intensity values were normalized applying quantile normalization and robust multi-array average (RMA) background correction. ANOVA was performed to identify differential expressed genes using a commercial software package SAS JMP10 Genomics, version 6, from SAS (SAS Institute, Cary, NC, USA). A false positive rate of α = 0.05 with false discovery correction was taken as the level of significance. Only genes with an adjusted *p*-value < 0.05 and a FC ≥ 1.5 or ≤−1.5 were considered differentially expressed, unless otherwise noted. All genes fulfilling the above criteria were subjected to gene set enrichment analysis using DAVID (version 6.8, https://david-d.ncifcrf.gov/)^72,73^. Here, GO analysis was performed using the categories GOTERM_BP_DIRECT and GOTERM_MF_DIRECT. Pathway analysis was performed using the KEGG database. Annotations with an adjusted *p*-value < 0.05 were considered significant.

Quantitative RT-PCR was analysed using a two-way ANOVA and Turkey’s multiple comparison test. Western blot experiments were analysed by densitometry of 5 independent experiments. One-tailed binomial test was calculated to compare observed and expected frequencies. A *p*-value of less than 0.05 was considered significant.

## Supplementary information

Supplementary Information

## Data Availability

The datasets generated during and/or analyzed during the current study are available in the GEO repository, GSE111123, https://www.ncbi.nlm.nih.gov/geo/query/acc.cgi?acc=GSE111123.
