# Supplementary material for: Methylglyoxal down-regulates the expression of cell cycle associated genes and activates the p53 pathway in human umbilical vein endothelial cells
Source: Sci Rep. 2019 Feb 4;9:1152. doi: 10.1038/s41598-018-37937-1 (PMC6362029; doi:10.1038/s41598-018-37937-1)
Supplement: Supplementary file 1 — Supplementary Information [file 41598_2018_37937_MOESM1_ESM.pdf]

Supplementary Information

Methylglyoxal down-regulates the expression of cell cycle associated genes and activates the p53 pathway in human umbilical vein endothelial cells

Jana D. Braun*[1], Diego O. Pastene[1], Annette Breedijk[1], Angelica Rodriguez[1], Björn B. Hofmann[1], Carsten Sticht[2], Elke von Ochsenstein[3], Heike Allgayer[3], Jacob van den Born[4], Stephan Bakker[4], Sibylle J. Hauske[1], Bernhard K. Krämer[1], Benito A. Yard[1] and Thomas Albrecht[1].

[1]Department of Nephrology, Endocrinology and Rheumatology, Fifth Department of Medicine, Medical Faculty Mannheim, University of Heidelberg, Mannheim, Germany
[2]Center of Medical Research, Medical Faculty Mannheim, University of Heidelberg, Mannheim, Germany [3] Department of Experimental Surgery - Cancer Metastasis, Medical Faculty Mannheim, University of Heidelberg, Mannheim, Germany,
[4]Department of Internal Medicine, University Medical Centre Groningen, Groningen, Netherlands

*Corresponding author: Jana Dorothea Braun, Department of Nephrology, Endocrinology and Rheumatology, Fifth Department of Medicine, Medical Faculty Mannheim, University of Heidelberg, Theodor-Kutzer-Ufer 1-3, 68167 Mannheim, phone +49 621 383 3212, fax +49 621 383 2610, jana.d.braun@outlook.de

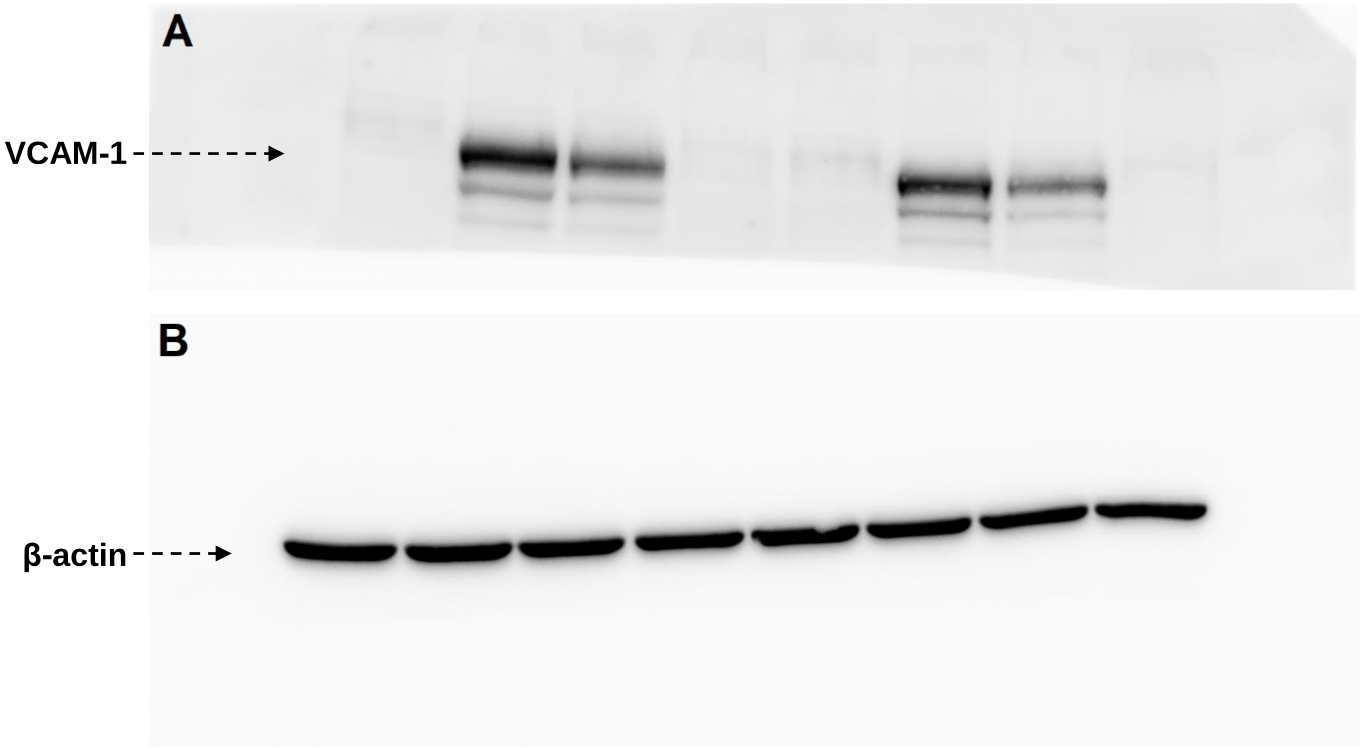

Supplementary Figure 1: Full length blots of figure 6A.

Full length blots of VCAM-1 (A) and β-actin (B). The arrows point to the relevant protein bands.

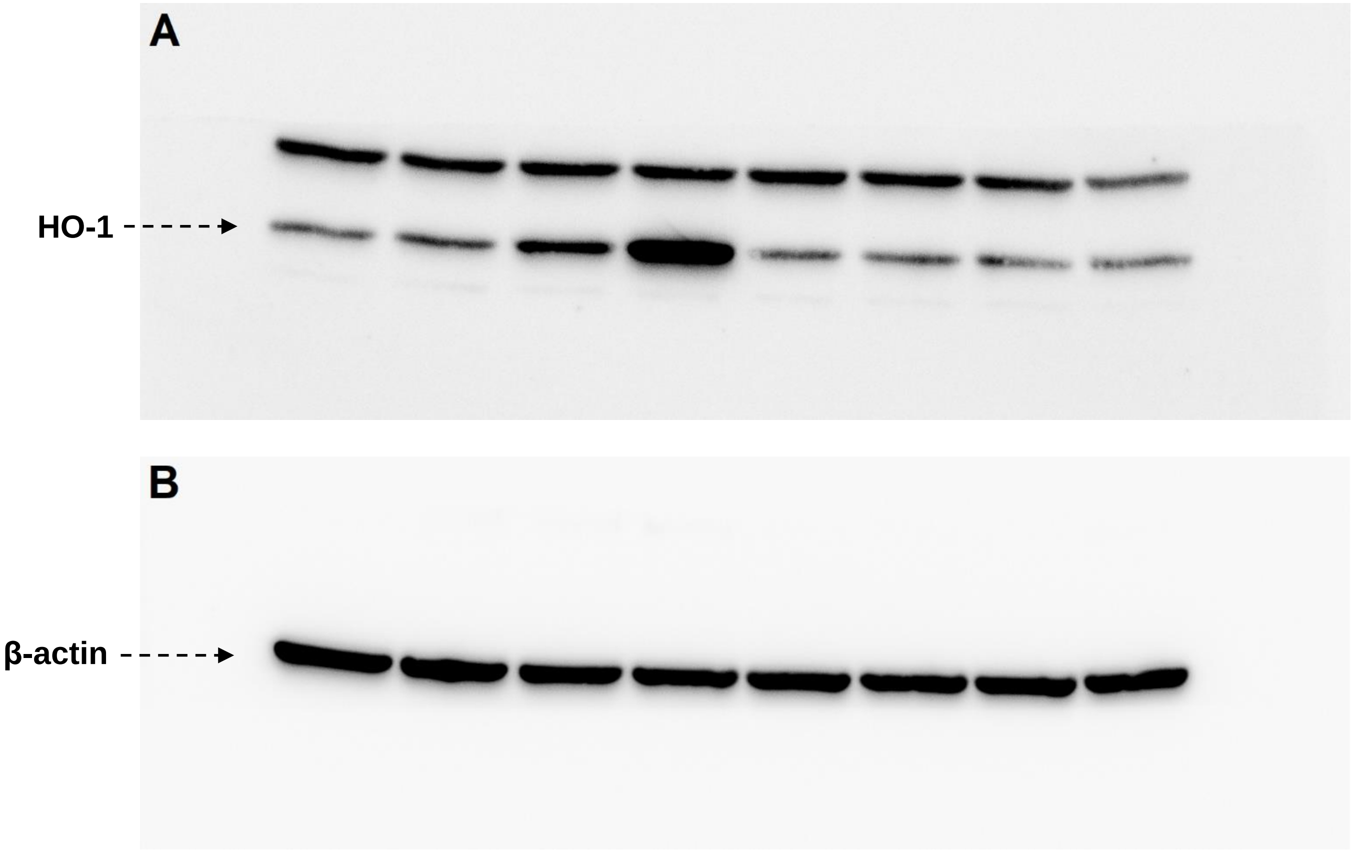

Supplementary Figure 2: Full length blots of figure 6B.

Full length blots of HO-1 (A) and β-actin (B). The arrows point to the relevant protein bands.

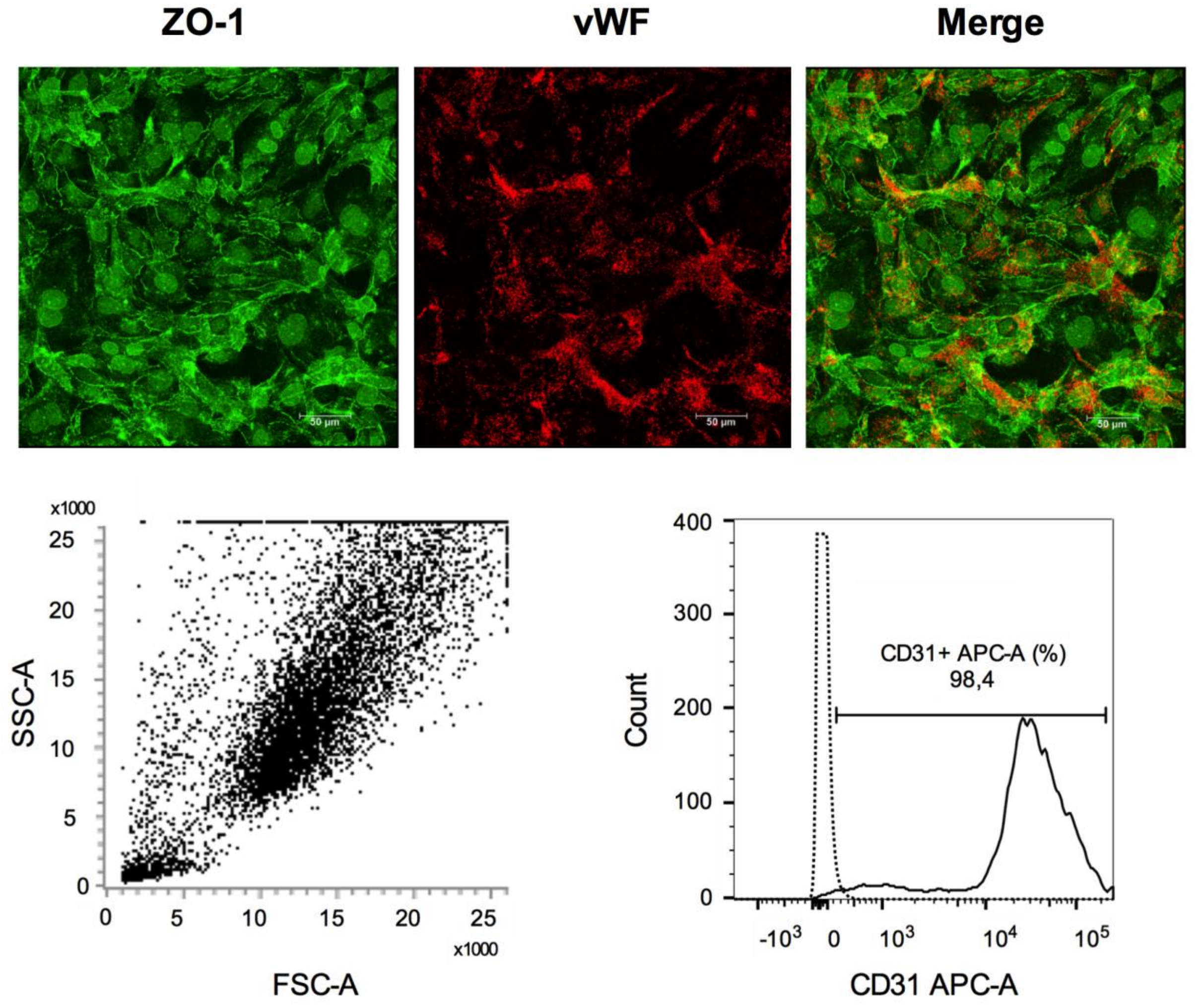

Supplementary Figure 3: Characterization of isolated endothelial cells from umbilical veins.

Isolated cells were grown until passage 2 and subsequently tested for endothelial cell purity. A: Cells grown on glass cover slips were stained for a general tight junction protein (Zonula occludens-1, ZO-1) to display cell boundaries (panel to the left) and the endothelial cell specific marker von Willebrand factor (vWF, panel in the middle). Note that in the merged panel (panel to the right) co-staining was seen in most if not all cells. B: FACS for the endothelial marker CD31 revealed a purity of more than 98%. Dotted line: histogram of the isotype-matched control, drawn line: histogram of CD31 staining.
